# Design and Rationale of the National Tunisian Registry of Heart Failure (NATURE-HF): Protocol for a Multicenter Registry Study

**DOI:** 10.2196/12262

**Published:** 2021-10-27

**Authors:** Leila Abid, Ikram Kammoun, Manel Ben Halima, Salma Charfeddine, Hedi Ben Slima, Meriem Drissa, Khadija Mzoughi, Dorra Mbarek, Leila Riahi, Saoussen Antit, Afef Ben Halima, Wejdene Ouechtati, Emna Allouche, Mehdi Mechri, Chedi Yousfi, Ali Khorchani, Omar Abid, Kais Sammoud, Khaled Ezzaouia, Imen Gtif, Sana Ouali, Feten Triki, Sonia Hamdi, Selim Boudiche, Marwa Chebbi, Mouna Hentati, Amani Farah, Habib Triki, Houda Ghardallou, Haythem Raddaoui, Sofien Zayed, Fares Azaiez, Fadwa Omri, Akram Zouari, Zine Ben Ali, Aymen Najjar, Houssem Thabet, Mouna Chaker, Samar Mohamed, Marwa Chouaieb, Abdelhamid Ben Jemaa, Haythem Tangour, Yassmine Kammoun, Mahmoud Bouhlel, Seifeddine Azaiez, Rim Letaief, Salah Maskhi, Aymen Amri, Hela Naanaa, Raoudha Othmani, Iheb Chahbani, Houcine Zargouni, Syrine Abid, Mokdad Ayari, Ines ben Ameur, Ali Gasmi, Nejeh ben Halima, Habib Haouala, Essia Boughzela, Lilia Zakhama, Soraya ben Youssef, Wided Nasraoui, Mohamed Rachid Boujnah, Nadia Barakett, Sondes Kraiem, Habiba Drissa, Ali Ben Khalfallah, Habib Gamra, Salem Kachboura, Leila Bezdah, Hedi Baccar, Sami Milouchi, Wissem Sdiri, Skander Ben Omrane, Salem Abdesselem, Alifa Kanoun, Karima Hezbri, Faiez Zannad, Alexandre Mebazaa, Samir Kammoun, Mohamed Sami Mourali, Faouzi Addad

**Affiliations:** 1 Société Tunisienne De Cardiologie Et De Chirurgie Cardiovasculaire Tunis Tunisia; 2 Hôpital Abderrahmen Mami-Ariana Ariana Tunisia; 3 Hôpital La Rabta 2 Tunis Tunisia; 4 Hôpital Des Forces De Sécurité Intérieure De La Marsa Tunis Tunisia; 5 Hôpital Regional Menzel Bourguiba Bizerte Tunisia; 6 Centre Hospitalier de Chambéry Chambéry France; 7 Hospital Lariboisière Paris France

**Keywords:** heart failure, acute heart failure, chronic heart failure, diagnosis, prognosis, treatment

## Abstract

**Background:**

The frequency of heart failure (HF) in Tunisia is on the rise and has now become a public health concern. This is mainly due to an aging Tunisian population (Tunisia has one of the oldest populations in Africa as well as the highest life expectancy in the continent) and an increase in coronary artery disease and hypertension. However, no extensive data are available on demographic characteristics, prognosis, and quality of care of patients with HF in Tunisia (nor in North Africa).

**Objective:**

The aim of this study was to analyze, follow, and evaluate patients with HF in a large nation-wide multicenter trial.

**Methods:**

A total of 1700 patients with HF diagnosed by the investigator will be included in the National Tunisian Registry of Heart Failure study (NATURE-HF). Patients must visit the cardiology clinic 1, 3, and 12 months after study inclusion. This follow-up is provided by the investigator. All data are collected via the DACIMA Clinical Suite web interface.

**Results:**

At the end of the study, we will note the occurrence of cardiovascular death (sudden death, coronary artery disease, refractory HF, stroke), death from any cause (cardiovascular and noncardiovascular), and the occurrence of a rehospitalization episode for an HF relapse during the follow-up period. Based on these data, we will evaluate the demographic characteristics of the study patients, the characteristics of pathological antecedents, and symptomatic and clinical features of HF. In addition, we will report the paraclinical examination findings such as the laboratory standard parameters and brain natriuretic peptides, electrocardiogram or 24-hour Holter monitoring, echocardiography, and coronarography. We will also provide a description of the therapeutic environment and therapeutic changes that occur during the 1-year follow-up of patients, adverse events following medical treatment and intervention during the 3- and 12-month follow-up, the evaluation of left ventricular ejection fraction during the 3- and 12-month follow-up, the overall rate of rehospitalization over the 1-year follow-up for an HF relapse, and the rate of rehospitalization during the first 3 months after inclusion into the study.

**Conclusions:**

The NATURE-HF study will fill a significant gap in the dynamic landscape of HF care and research. It will provide unique and necessary data on the management and outcomes of patients with HF. This study will yield the largest contemporary longitudinal cohort of patients with HF in Tunisia.

**Trial Registration:**

ClinicalTrials.gov NCT03262675; https://clinicaltrials.gov/ct2/show/NCT03262675

**International Registered Report Identifier (IRRID):**

DERR1-10.2196/12262

## Introduction

### Background

Heart failure (HF) has experienced epidemic growth and has become frequent worldwide. The prevalence of HF among the adult population in developed countries is 1%-2%; however, this rate rises to 10% or more among people aged over 70 [1]. According to recently published data, nearly 26 million people worldwide are living with HF; in particular, in Europe, this number is estimated to be 15 million [1,2]. The global health care cost related to HF is around US $108 billion/year [3]. Despite the development of therapeutic approaches, HF continues to account for a high mortality in up to 50% of patients within 5 years after diagnosis [4]; in particular, one-quarter of patients die within 1 year of diagnosis [5]. Sudden death is the most common cardiovascular cause of death (45%) [6,7].

The frequency of HF in Tunisia is on the rise and has now become a public health concern. This is mainly attributed to an aging Tunisian population (Tunisia has one of the oldest populations in Africa as well as the highest life expectancy in the continent) and an increase in coronary heart disease and hypertension. However, no extensive data are available on demographic characteristics, prognosis, and quality of care of patients with HF in Tunisia (nor in North Africa). Furthermore, the data pertaining to European and American populations cannot be extrapolated to the Tunisian population because of differences in demographics, diagnostic algorithm employed, and therapeutic strategies applied for patients with HF. Therefore, a multicenter observational study focusing on the demographic, prognostic, and therapeutic features of HF in Tunisia is essential. Data from such a study will help us to assimilate our practices, and then to harmonize them in order to optimize the management of patients and to assess the morbidity and mortality of HF as well as the degree of adherence of practitioners to international recommendations in the treatment of this pathology.

### Registry Objectives

The National Tunisian Registry of Heart Failure (NATURE-HF) describes the epidemiological profile of acute and chronic HF cases in Tunisia to evaluate their mobi-mortality over 1 year of follow-up. The morbidity of HF is defined as rehospitalization within 1 year of cardiac failure or flare-up occurring after inclusion of the patient, whereas mortality is defined as occurrence of cardiovascular death (sudden death, sudden cardiac arrest, refractory HF, stroke) or death from any cause (cardiovascular and noncardiovascular).

Several secondary endpoints are defined, including evaluation of (1) cardiovascular death over 1 year of follow-up, (2) all causes of death on a 1-year follow-up, (3) re-admission rate for cardiac insufficiency, (4) adherence of physicians to the European recommendations on medical and interventional therapies, (5) impact of HF on patient’s health, and (6) determination of the predictors of cardiovascular mortality.

## Methods

### Study Design and Patient Enrollment

This is a national, observational, longitudinal, multicenter registry study with a follow-up period of 13 months (1 month of inclusion and 12 months of clinical follow-up and exploratory analysis).

We included 1700 patients with HF, outpatients with chronic HF, and those hospitalized for acute HF. The diagnosis of HF is at the discretion of investigators. All included patients should provide written informed consent.

The exclusion criteria were as follows: estimated life expectancy less than 12 months for extracardiac pathology, isolated right HF, pregnancy, end-stage or severe renal insufficiency with creatinine clearance less than 15 mL/minute, undergoing hemodialysis, cardiac surgery scheduled within 3 months, and congenital heart disease.

Any violation of the protocol (eg, included but actually not eligible as per the selection criteria) will be informed to the Steering Committee which will decide on whether or not to exclude the patient concerned.

### Sample Size and Data Collection

Patients eligible according to aforementioned inclusion criteria will be selected at the cardiology consultation level or during cardiology or emergency hospitalization. As much as 250 cardiologists (from both public and liberal sectors) will participate in patient selection. Patient inclusion will occur consecutively until the end of the inclusion period. Suitable patients will be selected and requested to sign an informed consent form, confirming their agreement to participate in the study. A detailed questionnaire will then be administered to eligible patients and clinical, biological, and exploratory data will be collected.

The inclusion began on October 2, 2017 (duration 1 month). A regular follow-up was done, wherein patients must visit the cardiology clinic 1, 3, and 12 months after inclusion into the study. This follow-up is provided by the investigator.

Given the observational nature of the NATURE-HF study, no specific treatment or intervention is planned for the management of HF. Patients should be cared for according to the usual medical procedures.

All data are collected via the DACIMA Clinical Suite web interface [8]. The platform complies with international standards FDA 21 CFR part 11 (Food and Drug Administration 21 Code of Federal Regulations part 11), HIPPA (Health Insurance Portability and Accountability Act), ICH (International Conference on Harmonisation), MedDRA (Medical Dictionary for Regulatory Activities), Health Canada, and Tunisian regulations.

### Statistical Analysis

The DACIMA Clinical Suite platform enables the collection of online data and their extraction in SAS or SPSS format. Statistical analysis will be performed with SAS software (University Edition; SAS Institute). The statistical analysis is exploratory, involving the calculation of 95% CI.

The data will be described for the whole analyzed population. Continuous quantitative variables (eg, age, weight, left ventricular ejection fraction, biological parameters) will be described by the number of patients documented, mean, median, SD, and extreme values. Categorical variables will be described by the number and percentage of each category.

The normality of the continuous quantitative variables will be verified with the Shapiro–Wilk test. If the hypothesis of normality is rejected, then a simple transformation will be made on the variable to normalize it. If this transformation succeeds in normalizing the variable, then the analysis will be performed on the transformed variable. Otherwise nonparametric tests will be used or possibly an analysis on the quartiles or quintiles will be performed.

Statistical tests will be bilateral with a statistically significant threshold of 5%. An analysis of variance will be performed for the quantitative variables (parametric test for the variables that follow a normal distribution, and nonparametric test in other cases) and a chi-square test will be realized for the categorical variables. The Yates correction or Fisher exact test will be used if the validity conditions for the chi-square test are not met (theoretical number <5).

The sample size (both power and sample) is estimated by the SPSS 2008 software (IBM). To estimate the number of patients to be included in this study, a range of 95% CI (5% significance threshold) is set as the primary endpoint. The impact of this criterion is estimated at 35%, based on the US registry OPTIMIZE-HF [9], which found a hospital mortality ranging from 2.2% to 4.1%, a global mortality ranging between 6.5% and 9.1%, and an overall incidence of all events evaluated (rehospitalization or death) ranging from 35.3% to 36.6%.

Considering these data, the number of patients to be included in the study would be 1436. However, by predicting a 5% missing data rate and a 10% loss to follow-up, the sample size to be selected will be 1700 patients.

The intermediate analyses will be carried out after formulating the statistical analysis plan.

### Data Review

The Data Review Committee includes the Coordinators of the Steering Committee of the study as well as the scientific team of DACIMA Consulting. The purpose of the Data Review Committee is to respond to specific queries in data management, and to validate the statistical procedures developed in the final statistical analysis plan. The Data Review Committee will also be responsible for validating subsequent publications.

### Expected Implications

The NATURE-HF is the first large-scale investigation to clarify the contemporary demographic data, management, and outcomes of patients with HF.

### Oversight and Leadership

The protocol of the NATURE-HF registry has been approved by the Tunisian Society of Cardiology and Cardiovascular Surgery. The NATURE-HF study has been registered in ClinicalTrials.gov (trial registration number NCT03262675).

### Study Sponsorship

The NATURE-HF registry is sponsored by the Tunisian Society of Cardiology and Cardiovascular Surgery.

## Results

A total of 95 cardiologists included 1700 patients in the registry with a 1-year follow-up period. All patients provided written informed consent. Patients were officially enrolled in NATURE-HF only if they were aged 20 years or older.

The NATURE-HF registry does not impose any specific intervention. Treatment of patients follows the usual recommendations for the management of HF. Clinical events occurring during the follow-up will be collected in order to evaluate the judgment criteria planned in the protocol. However, at the end of the study, a detailed report of the clinical events will be prepared by the STCCCV (Société Tunisienne De Cardiologie Et De Chirurgie Cardiovasculaire [Tunisian Society of Cardiology and Cardiovascular Surgery]) and the document provided to the National Center for Pharmaco-Vigilance.

## Discussion

HF is a major health concern worldwide and one of the most important causes of hospitalization [10]. Acute HF is responsible for over 1 million hospitalizations each year in the United States, with a similar number also being reported in Europe [11]. These hospitalizations are responsible for an increased economic burden and are associated with high mortality rates, up to 20% in the 6 months following hospital discharge [12]. HF management is challenging given the heterogeneity of the patient population, absence of an universally accepted definition, incomplete understanding of its pathophysiology, and lack of evidence-based guidelines. Most patients appear to respond well to initial therapies consisting of loop diuretics and vasoactive agents [13,14]. However, these treatments fail to decrease postdischarge mortality and re-admission rates. In the last few years, many drugs have been evaluated to treat HF; unfortunately, results were disappointing in terms of efficacy and safety [15-22].

This large, contemporary longitudinal study of Tunisian patients with HF will provide a unique opportunity to answer many clinical questions. The NATURE-HF study is important in several respects. First, systematic observational and outcomes data can be generated from this registry study, which is especially valuable given that previous data for Tunisian patients with HF are limited. Second, treatment of HF changes dramatically, and thus HF management needs to be evaluated in real-world studies. Third, the NATURE-HF study provides a good opportunity to compare treatment and response variation among HF populations in Africa for comparison with different countries and evaluate adherence to recent guidelines.

The NATURE-HF study will fill a significant gap in the dynamic landscape of HF care and research. It will provide unique and necessary data on the management and outcomes of patients with HF. This study will yield the largest contemporary longitudinal cohort of patients with HF in Tunisia.

## Abbreviations

FDA: Food and Drug Administration
HF: heart failure
HIPPA: Health Insurance Portability and Accountability Act
ICH: International Conference on Harmonisation
MedDRA: Medical Dictionary for Regulatory Activities
STCCCV: Société Tunisienne De Cardiologie Et De Chirurgie Cardiovasculaire (Tunisian Society of Cardiology and Cardiovascular Surgery)
